# Endovascular Thrombectomy preceded by intravenous Alteplase versus endovascular Thrombectomy alone in Han Chinese patients treated for acute ischemic stroke with large vessel occlusion: a single-center retrospective analysis

**DOI:** 10.1186/s12883-021-02401-7

**Published:** 2021-09-28

**Authors:** Ruodong Han, Bowen Li, Yajie Yue, Guozhu Wu, Xiuxia Yan

**Affiliations:** Department of Critical Care Medicine, People’s Hospital of Bozhou, Bozhou Hospital Affiliated to Anhui Medical University, No. 616, Duzhong Road, Bozhou City, 236800 Anhui Province China

**Keywords:** Alteplase, Intracranial hemorrhage, Modified Rankin scale score, Recanalization, Stroke, Thrombectomy

## Abstract

**Background:**

The American Heart Association/ American Stroke Association and the Chinese Stroke Association guidelines are recommending intravenous alteplase intervention before endovascular thrombectomy if patients are eligible to do so but the benefits of endovascular thrombectomy are different in Chinese patients with stroke than those of the white patients. The objective of the study was to compare outcomes of patients with acute ischemic stroke treated with endovascular thrombectomy with intravenous alteplase against those treated with endovascular thrombectomy alone.

**Methods:**

A report is a retrospective analysis of comparing demographics, imaging, clinical and adverse outcomes in the Han Chinese patient who underwent mechanical thrombectomy for acute ischemic stroke with large vessel occlusion, with or without preceding intravenous alteplase administration. Patients with terminus and non-terminus intracranial occlusions and ≤ 2 points neurologic deficit underwent endovascular thrombectomy preceded by 0.9 mg/ kg intravenous alteplase (ET cohort, *n* = 184) and those who had contra-indication for intravenous alteplase were treated with endovascular thrombectomy alone (EA cohort, *n* = 141).

**Results:**

The most common procedural complications were embolization into new territory (*p* = 0.866) and uneventful artery vasospasm (*p* = 0.712). Insignificant differences were reported for any procedural complications (*p* = 0.991), imaging outcomes, the modified Rankin scale score (*p* = 0.663), and death (28 vs. 24, *p* = 0.761) within 90 days between patients of both cohorts. At the discharge of the hospital, the National Institutes of Health Stroke Scale scores of patients of the ET cohort were lower than those of the EA cohort (8.58 ± 3.79 vs. 10.23 ± 4.97, *p* = 0.003). The Barthel Index of survivors at 90 days after endovascular thrombectomy was higher for patients of the ET cohort than those of the EA cohort (87.47 ± 12.58 vs. 84.01 ± 13.47, *p* = 0.032). The most common adverse effect was asymptomatic intracranial hemorrhage (*p* = 0.297). Insignificant differences were reported for adverse effects after thrombectomy between survivors of both cohorts.

**Conclusions:**

Outcome measures in Han Chinese patients with acute ischemic stroke treated with endovascular thrombectomy alone were statistically the same as those treated with endovascular thrombectomy plus intravenous alteplase.

**Level of evidence:**

Iii

**Technical efficacy stage:**

4.

## Background

Stroke is the major cause of death in China [1] and the prevalence of stroke has risen in the Chinese population in recent years [2]. These have imposed a heavy burden on the healthcare system of China [1]. Endovascular thrombectomy is used as a part of the standard treatment(s) in patients of acute ischemic stroke in the anterior cerebral circulation due to large-vessel occlusion [3]. Alteplase is generally administered intravenously in cases of endovascular thrombectomy [2]. The exact role of alteplase before and during endovascular thrombectomy is uncertain. Probably, alteplase increases reperfusion of the ischemic area and dissolves thrombi after endovascular thrombectomy [4]. Pooled analyses [5, 6], a meta-analysis [7], and the DEVT trial [8] suggest equivalent effects of endovascular thrombectomy alone and combined treatments of endovascular thrombectomy with intravenous alteplase intervention. However, a randomized trial [2], a meta-analysis [9], and a SKIP trial [10] show the superior effect of endovascular thrombectomy combined with intravenous alteplase intervention compared to endovascular thrombectomy alone. Intravenous alteplase intervention with endovascular thrombectomy is effective for emergency revascularization [11] but may have changes of coagulation abnormalities and hemorrhagic complications [12]. Further, intravenous alteplase intervention has a poor response as compared to the endovascular thrombectomy in the management of stroke in patients with large vessel occlusion. In most cases, intravenous alteplase intervention induces partial recanalization, since stroke patients often have a large thrombus burden. Thus, the presence of a proximal arterial occlusion does not necessarily lead to alteplase intervention failure, since some degree of recanalization can occur even with large thrombi [13]. The American Heart Association/ American Stroke Association [14] and the Chinese Stroke Association guidelines [15] are recommending intravenous alteplase intervention before endovascular thrombectomy if patients are eligible to do so but the beneficial effects of endovascular thrombectomy are different in Chinese patients with stroke than those of white patients because more numbers of cases with intracranial atherosclerosis among Chinese patients with stroke [16]. Therefore, there is uncertainty about the favorable and unfavorable effects of intravenous administration of alteplase before endovascular thrombectomy in Chinese patients with acute ischemic stroke because the currently available data are inconclusive regarding the efficacy of mechanical thrombectomy alone or in combination with intravenous thrombolysis.

The objectives of the retrospective study were to compare procedural complications, imaging outcomes, the ischemic stroke conditions, functional outcome measures, health status, adverse effects, and death of Han Chinese patients treated with endovascular thrombectomy with intravenous alteplase against those treated with endovascular thrombectomy alone for acute ischemic stroke with large vessel occlusion.

## Methods

### Inclusion criteria

Patients 18 years and above of acute ischemic stroke who had terminus and non-terminus intracranial occlusions of the internal carotid artery and/ or occlusions of the first or proximal second segment of the middle cerebral artery (the results as per the computed tomographic angiography) and had a neurologic deficit 2 points or less (according to the modified Rankin scale score) were included in the analysis.

### Exclusion criteria

Patients who had the modified Rankin scale score for disability more than 2 before stroke were excluded from the analysis.

### Sample size calculation

The sample size was calculated based on the assumption that endovascular thrombectomy with/ without intravenous alteplase intervention would be improved the modified Rankin scales for disability of at least 10% of patients, 80% power calculation (*β* = 0.2), two-sided type-I error (*α* = 0.05), and at 95% of confidence level. The sample size (minimum number of patients required in each cohort) was 105.

### Cohorts

A total of 184 patients underwent endovascular thrombectomy using AngioJet AVX Thrombectomy Catheter (Boston Scientific Corporation, Boston, MA, USA) preceded by 0.9 mg/ kg intravenous alteplase (10% bolus and 90% infusion throughout 1 h) administered within 4.5 h after symptom onset (EA cohort). A total of 141 patients have contra-indication for intravenous alteplase according to the American Heart Association-American Stroke Association guidelines [17] (serious head trauma, prior stroke, hemorrhagic coagulopathy, or receiving anticoagulant medications), so they underwent endovascular thrombectomy only (ET cohort).

### Data collection

Data regarding procedural complications, imaging outcomes before thrombectomy and after endovascular thrombectomy, the National Institutes of Health Stroke Scale score before endovascular thrombectomy, 1-day after, and one week after of endovascular thrombectomy (or discharge of hospital), the modified Rankin scale score before endovascular thrombectomy and at 90 days after endovascular thrombectomy, death of patients within 90 days after endovascular thrombectomy, the Barthel Index at 90 days after endovascular thrombectomy, health status at 90 days after endovascular thrombectomy, recanalization at 3 days after endovascular thrombectomy, the final lesion volume at the discharge of hospital, and adverse effects within 90 days after endovascular thrombectomy were retrospectively collected from the patients’ records of institutes.

### Ischemic stroke condition

The severity of the ischemic stroke condition was assessed by the National Institutes of Health Stroke Scale score. It was in the range of 0–42. 0–6: mild symptoms, 7–14: moderate symptoms, and ≥ 15 severe symptoms [18].

### Functional outcomes

#### The modified Rankin scale score

It was used for the assessment of the severity of the disability. 0: no disability, 1: no significant disability but has symptoms, 2: slight disability to perform the daily activity, 3: able to walk without assistance or require some help (moderate disability), 4: unable to walk without assistance, and 5: requires constant nursing care for daily activity [19].

### Imaging outcomes

#### The Alberta stroke program early computed tomography score

It is a measure of the extent of early cerebral ischemia. Scores range from 0 to 10, with higher scores indicating early ischemic changes are less [2].

#### Reperfusion

The extended Thrombolysis in Cerebral Infarction score was preferred for evaluation of reperfusion of the occluded vascular territory using digital subtraction angiography. The score is ranged from 0 to 3. 0: no antegrade reperfusion, 1: limited antegrade reperfusion, 2a: less than 50% antegrade reperfusion, 2b: 50% or more but less than 90% antegrade reperfusion, 2c: 90% or more antegrade reperfusion, and 3: complete (100%) antegrade reperfusion [20].

#### Recanalization

The modified Arterial Occlusive Lesion score as per the computed tomographic angiography results was used to defined recanalization. The score ranges from 0 to 3. 0: No recanalization, 1: partial recanalization without passage of contrast agent, 2: partial recanalization with the passage of contrast agent, and 3: complete recanalization [11].

#### The final lesion volume

The final lesion volume was measured by the non-contrast computed tomography. Automatic measurement software was used for the measurement of volume.

### Quality of life

#### The Barthel index

It is the 10-item scale of 10 basic activities of self-care and mobility. The activities included for consideration were urinary bladder management, bowel management, toilet management, bathing, dressing, grooming, feeding, walking, transfer, and ascending and descending staircases. Each item has a 0 to 10 scale. If an individual item was performed by patients without assistance of anything, considered as maximum 10, and the item performed using help was considered as 0. The total score of the Barthel Index was 100. Score 0 indicating severe disability that interferes with daily activities and a score of 95 or more indicating no disability interferes with daily activities [21].

#### Health status

Health status was accessed using the 5-Dimension 5-Level Self-Report Questionnaire. Five dimensions were mobility, self-care, usual activity, pain/ discomfort, and anxiety/ depression. Five levels were 1: normal, 2: slight problem, 3: moderate problem, 4: severe problem, 5: extreme problem. It is ranged from − 0.39 to 1. 0: death, 1: full health, and a negative value indicates a condition worse than death [22].

### Adverse effects

Heidelberg criteria were used for the evaluation of intracranial hemorrhage [23].

### Statistical analysis

InStat 3.0.1, GraphPad software, USA was used for statistical analysis. Unpaired *t*-test was performed for continuous variables and Fischer exact test or the Chi-square test of Independence was performed for constant variables. If the *p*-value was less than 0.05, the results were considered significant.

## Results

### Study population

**Fig. 1:**
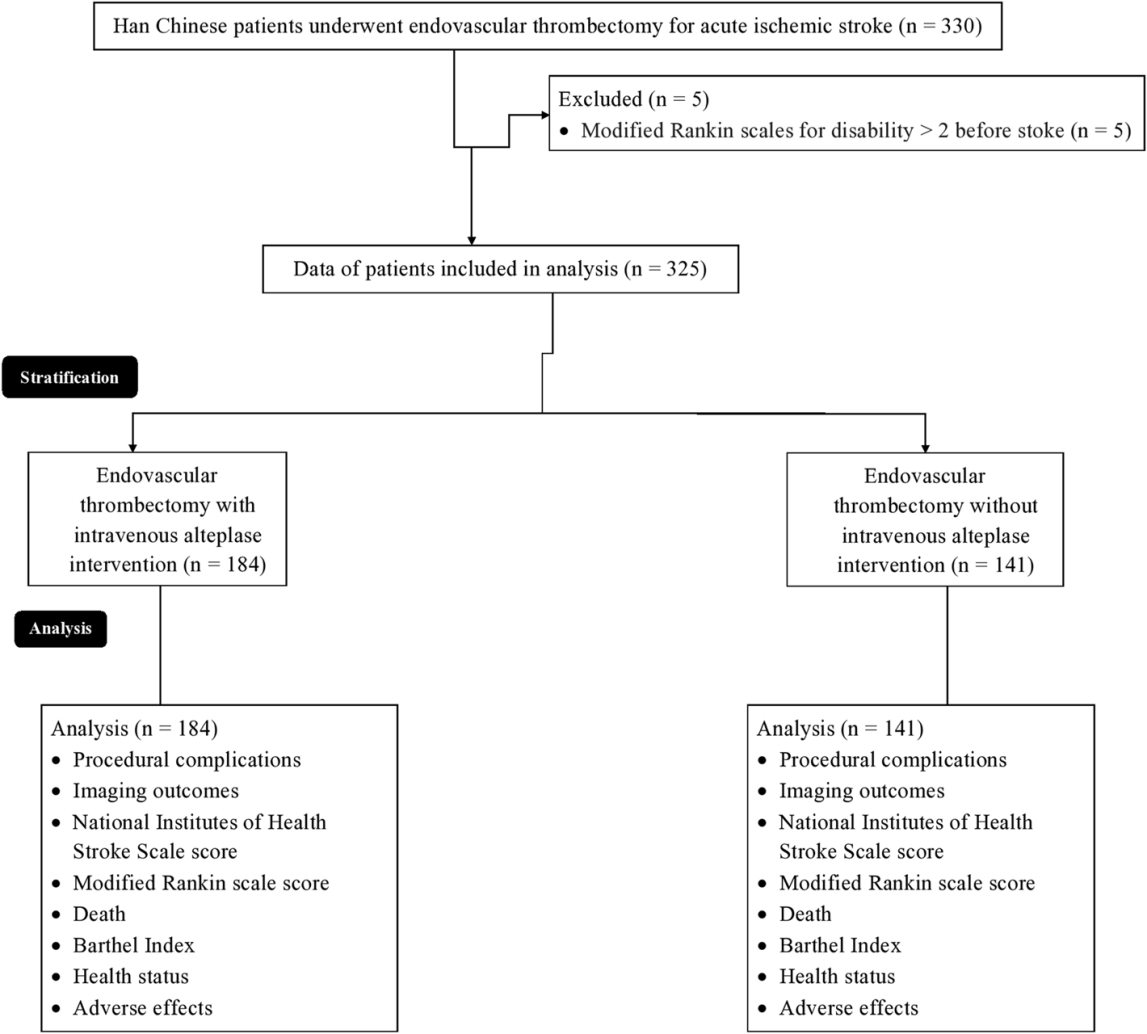
The flow diagram of management of acute ischemic stroke

From January 2, 2018, to August 11, 2019, a total of 330 Han Chinese patients underwent endovascular thrombectomy alone or with intravenous alteplase for management of the acute ischemic stroke at the People’s Hospital of Bozhou, Bozhou Hospital Affiliated to Anhui Medical University, Bozhou, Anhui, China. Among them, 5 patients had the modified Rankin scale score for disability more than 2 before stoke treatment(s). Therefore, these patients were excluded from the analysis. Data regarding imaging outcomes, the ischemic stroke condition, the functional outcomes, death of patients within 90 days after endovascular thrombectomy, health status, recanalization, the final lesion volume, and adverse effects within 90 days after endovascular thrombectomy of the total of 325 Han Chinese patients were retrospectively collected from the patients’ records of institutes and analyzed. The flow diagram for the treatment of acute ischemic stroke is presented in Fig. 1.

### Characteristics of patients

There were no significant differences for the clinical and demographical characteristics of patients before treatment(s) (for stroke) between cohorts (*p* > 0.05 for all). The details of the clinical and demographical characteristics of patients before treatment(s) (for stroke) are reported in Table 1. The reasons for intravenous alteplase treatment contraindications for the patients of the ET cohort are summarized in Table 2.

**Table 1:** The clinical and demographical characteristics of patients before treatment(s) (for stroke)

Parameters	Cohorts		Comparisons
	EA cohort	ET cohort	
Treatment(s)	Endovascular thrombectomy + intravenous alteplase intervention	Endovascular thrombectomy	
Numbers of patients who underwent treatment	184	141	*p*-value
Age (years)			
Minimum	58	59	0.231
Maximum	73	71	
Mean ± SD	64.12 ± 5.49	63.41 ± 5.01	
Sex			
Male	103 (56)	89 (63)	0.212
Female	81 (44)	52 (37)	
The National Institutes of Health Stroke Scale score			
Minimum	6	6	0.441
Maximum	22	22	
Median	14	14	
Mean ± SD	16.34 ± 4.60	13.94 ± 4.69	
Previous ischemic stroke	21 (11)	19 (13)	0.612
History of atrial fibrillation	71 (39)	49 (35)	0.489
Type-2 diabetic	32 (17)	28 (20)	0.568
Hypertensive	101 (55)	82 (58)	0.574
Hypercholesterolemia	8 (4)	7 (5)	0.796
Peripheral artery disease	3 (2)	1 (1)	0.636
Smoking			
No smoker	147 (80)	108 (77)	0.768
Previous smoker	35 (19)	31 (22)	
Current smoker	2 (1)	2 (1)	
The modified Rankin scale score			
0	120 (65)	82 (58)	0.427
1	45 (24)	41 (29)	
2	19 (10)	18 (13)	
The Alberta Stroke Program Early Computed Tomography Score	8.15 ± 1.85	8.11 ± 1.86	0.847
Systolic blood pressure (mmHg)	145 ± 11	143 ± 12	0.119
Blood glucose (mg/ dL)	129 ± 11	127 ± 15	0.167
Reasons for stroke			
Cardio-embolism	79 (43)	61 (43)	0.973
Intracranial atherosclerosis	15 (8)	13 (9)	
Ipsilateral extracranial internal carotid artery obstruction	21 (11)	17 (12)	
Unspecified	69 (38)	50 (35)	
Intracranial artery occlusion location			
Intracranial internal carotid artery	61 (33)	41 (29)	0.681
M1 segment of middle cerebral artery	95 (52)	75 (53)	
M2 segment of middle cerebral artery	28 (15)	25 (18)	

Constant variables are demonstrated as frequency (percentages) and continuous and ordinal variables are demonstrated as mean ± standard deviation (SD)

Unpaired *t*-test (for continuous and ordinal variables) and Fischer exact test or the Chi-square test of Independence (for constant variables) were performed for statistical analysis

Results were significant if *p*-value was < 0.05

**Table 2:** Summarizing the reasons of intravenous alteplase treatment contraindications

Reason	Numbers of patients
Serious head trauma	35 (25)
Hemorrhagic coagulopathy	37 (26)
Receiving anticoagulant medications	69 (49)
Total	141 (100)

Variables are demonstrated as frequency (percentages)

### Procedural complications

The most common procedural complications were embolization into a new territory and uneventful artery vasospasm. There were no significant differences for the any procedural complications between both cohorts (*p* = 0.991). The details of procedural complications are reported in Table 3.

**Table 3:** Procedural complications

Complications	Cohorts		Comparisons
	EA cohort	ET cohort	
Treatment	Endovascular thrombectomy + intravenous alteplase intervention	Endovascular thrombectomy	
Numbers of patients who underwent treatment	184	141	*p*-value
Vessel dissection	6 (3)	3 (2)	0.539
Contrast extravasation	5 (3)	5 (4)	0.669
Embolization into a new territory	22 (12)	16 (11)	0.866
Femoral access complications	2 (1)	1 (1)	0.725
Uneventful artery vasospasm	21 (11)	18 (13)	0.712
Any procedural complication	56 (30)	43 (30)	0.991

Variables are demonstrated as frequency (percentages)

An unpaired *t*-test was performed for statistical analysis

Results were significant if *p*-value < 0.05

Patients might have more than one complication

### Time metrics

Time from door to the interpretation of computed tomography angiography for patients of the EA cohort was 17.18 ± 1.15 min and that for patients of the ET cohort was 16.95 ± 1.51 (*p* = 0.119). The onset time to groin puncture for patients of the EA cohort was 53.15 ± 5.8 min and that for patients of the ET cohort was 52.31 ± 6.1 min (*p* = 0.207). For patients of the EA cohort, the onset time to intravenous alteplase treatment decision was 90.15 ± 28.5 min, and door to intravenous alteplase treatment time was 54.52 ± 2.15 min.

### Outcome measures

#### The ischemic stroke conditions

**Fig. 2:**
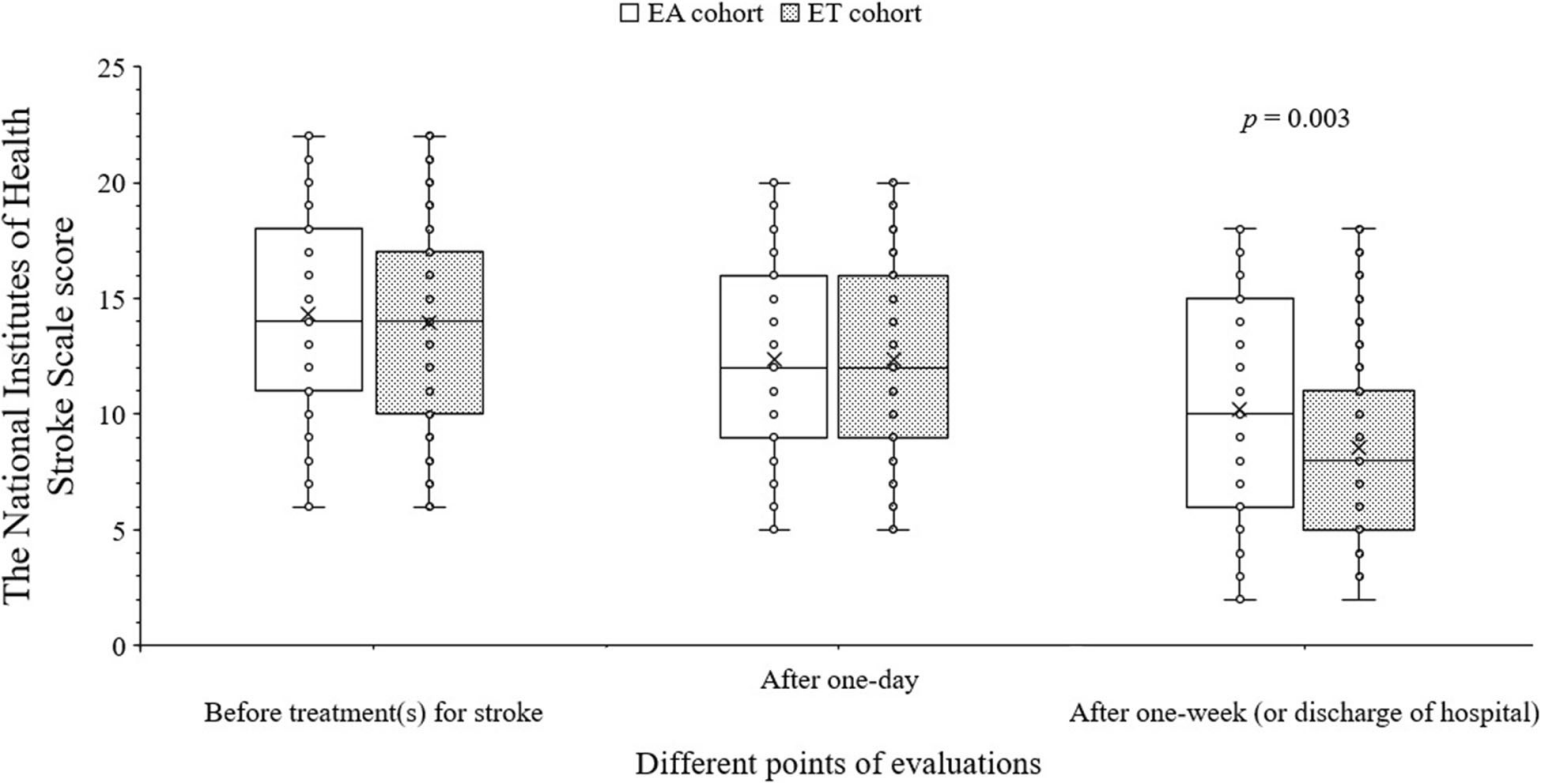
The National Institutes of Health Stroke Scale scores of patients. 0–6: mild symptoms, 7–14: moderate symptoms, and ≥ 15 severe symptoms

There was no significant difference for the National Institutes of Health Stroke Scale scores of survivors at one day after endovascular thrombectomy between both cohorts (12.39 ± 4.20 (5–20; median: 12) vs. 12.36 ± 4.30 (5–20; median: 12), *p* = 0.947). However, one week after endovascular thrombectomy or discharge from hospital, the National Institutes of Health Stroke Scale scores of patients of the ET cohort were lower than those of the EA cohort (8.58 ± 3.79 (2–18; median: 8) vs. 10.23 ± 4.97 (2–18; median: 10), *p* = 0.003). The details of the National Institutes of Health Stroke Scale scores of survivors are reported in Fig. 2.

#### Functional and imaging outcome measures

**Fig. 3:**
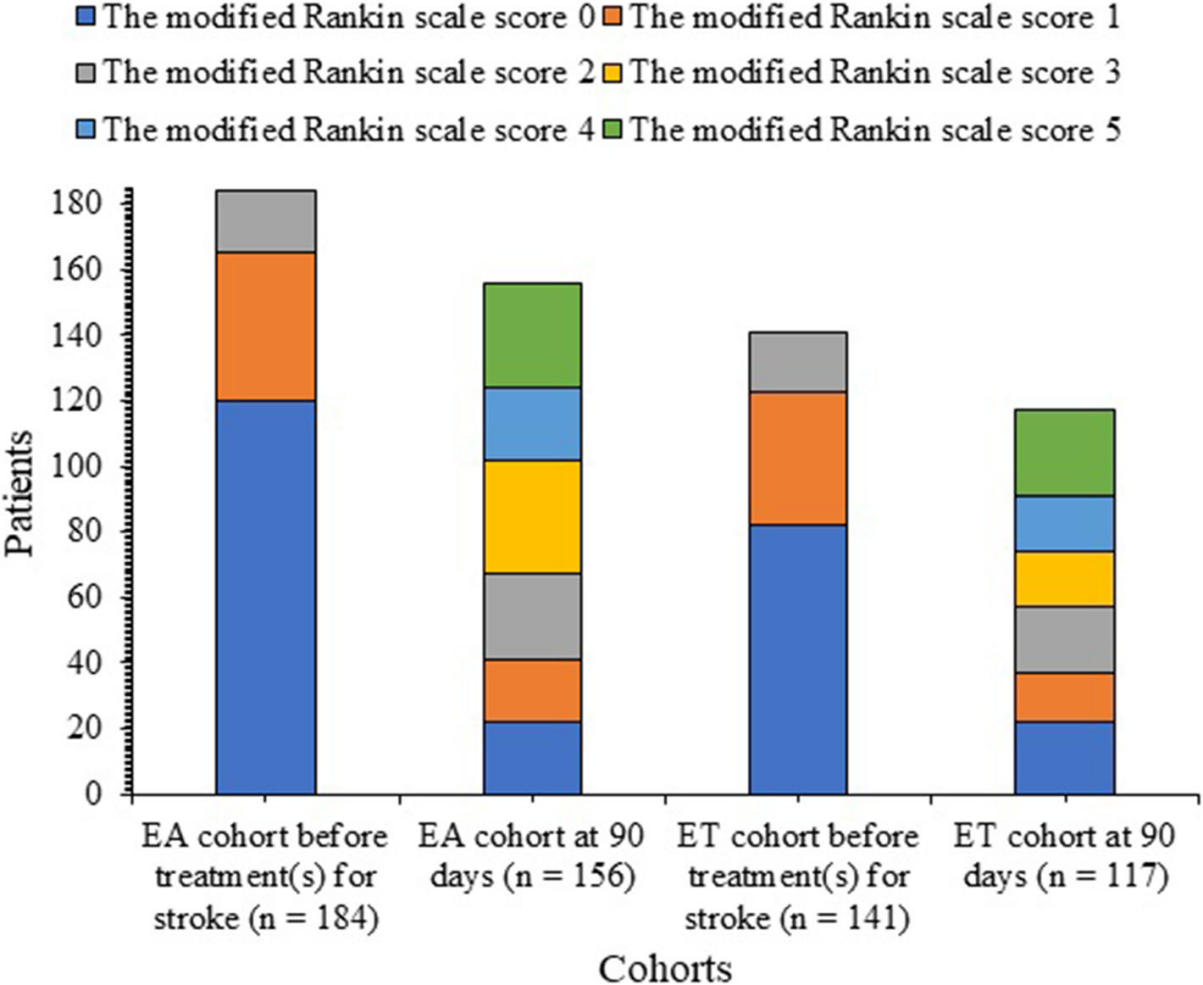
The modified Rankin scale score of survivors at 90 days. 0: no disability, 1: no significant disability but has symptoms, 2: slight disability to perform the daily activity, 3: able to walk without assistance or require some help (moderate disability), 4: unable to walk without assistance, and 5: requires constant nursing care for daily activity

There was no significant difference for the modified Rankin scale score of survivors at 90 days after endovascular thrombectomy between both cohorts (*p* = 0.663, Fig. 3). There were no significant differences for the reperfusion of survivors at one day after endovascular thrombectomy between both cohorts (*p* = 0.124), the recanalization of survivors at 3 days after endovascular thrombectomy between both cohorts (*p* = 0.384), and the final lesion volume of survivors at the discharge of hospital (42.15 ± 13.18 mL vs. 40.22 ± 11.18 mL, *p* = 0.203).

### Quality of life

#### The Barthel index

**Fig. 4:**
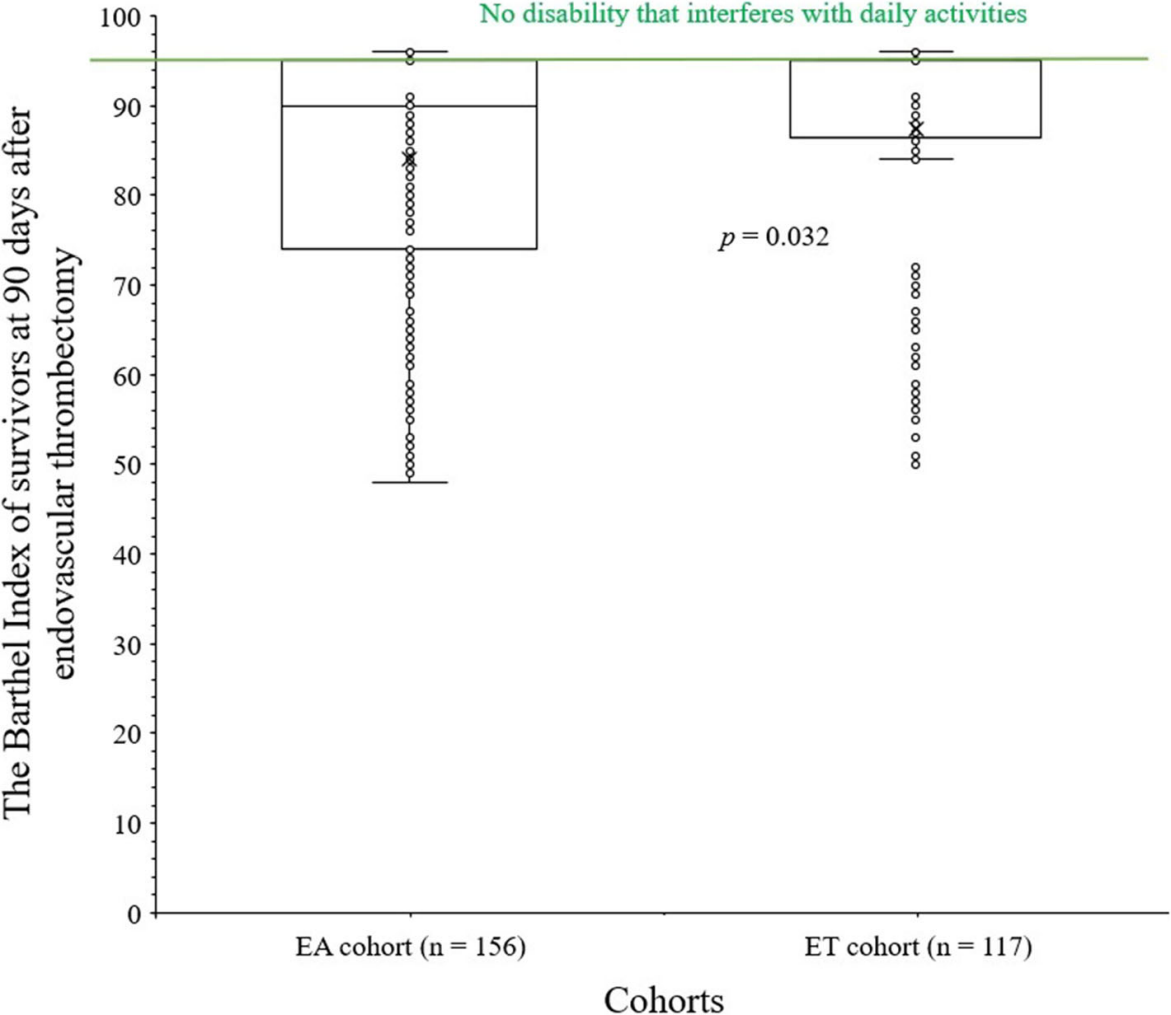
The Barthel Index score of survivors at 90 days after endovascular thrombectomy

The Barthel Index of survivors at 90 days after endovascular thrombectomy was higher for patients of the ET cohort than those of the EA cohort (87.47 ± 12.58 vs. 84.01 ± 13.47, *p* = 0.032). The details of the Barthel Index score of survivors are reported in Fig. 4.

#### Health status

**Fig. 5:**
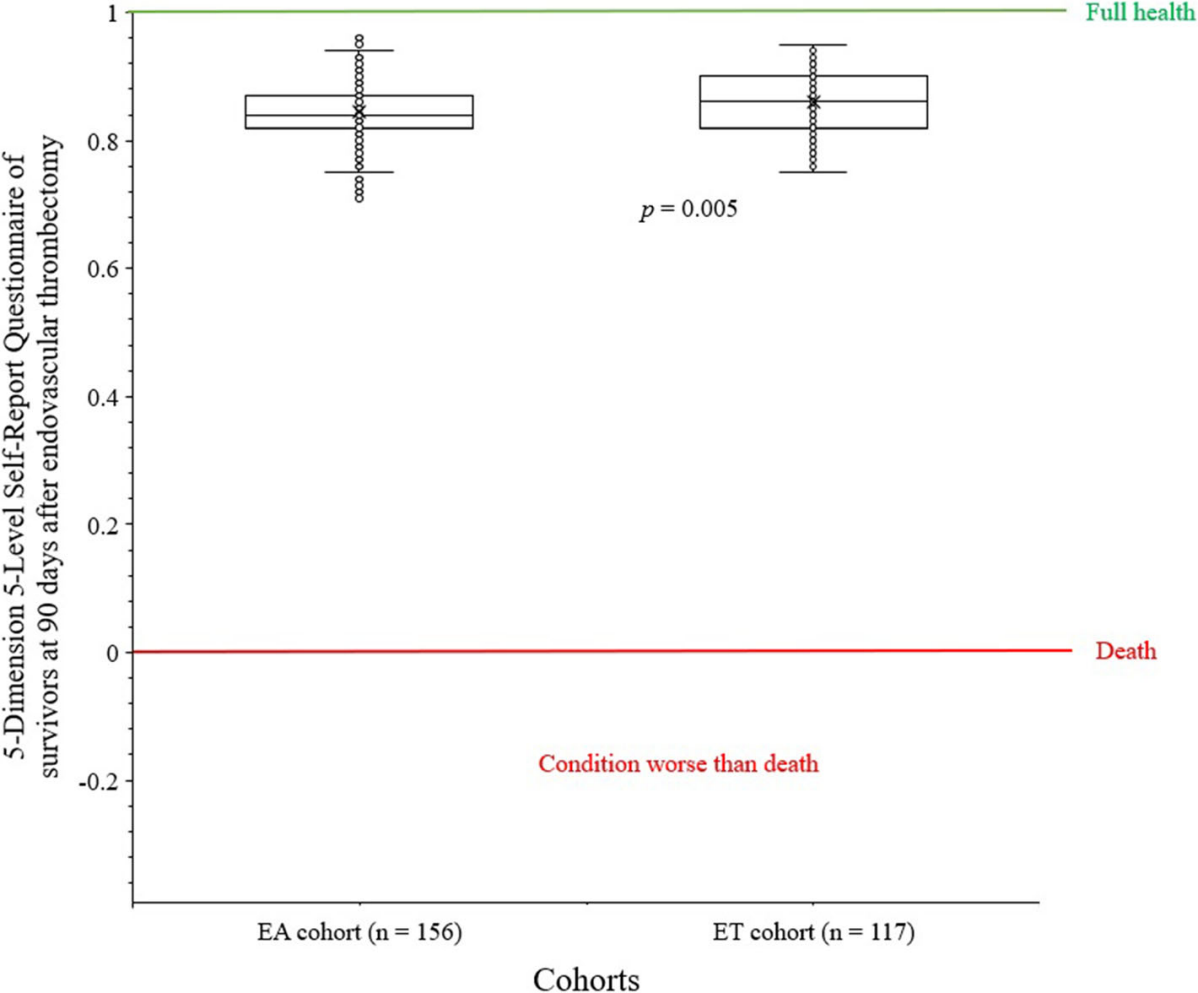
5-Dimension 5-Level Self-Report Questionnaire of survivors at 90 days after endovascular thrombectomy with/ without intravenous alteplase intervention. 0: death, 1: full health, and a negative value indicates a condition worse than death

5-Dimension 5-Level Self-Report Questionnaire of survivors at 90 days after endovascular thrombectomy was higher for patients of the ET cohort than those of the EA cohort (0.86 ± 0.04 vs. 0.85 ± 0.04, *p* = 0.005). The details of the 5-Dimension 5-Level Self-Report Questionnaire of survivors are reported in Fig. 5.

The details of all outcome measures after endovascular thrombectomy are reported in Table 4.

**Table 4:** Functional and imaging outcome measures after endovascular thrombectomy with/ without intravenous alteplase intervention

Outcome measure			Cohorts		Comparisons
			EA cohort	ET cohort	
Treatment(s)			Endovascular thrombectomy + intravenous alteplase intervention	Endovascular thrombectomy	*p*-value
Death within 90 days			28 (15)	24 (17)	0.761
Numbers of survivors with ≥95 the Barthel Index score at 90 days			75 (48)	63 (54)	0.392
Imaging outcomes	Successful reperfusion before thrombectomy		7 (4)	8 (6)	0.438
	Reperfusion of survivors after one-day	0	13 (7)	15 (11)	0.124
		1	02 (1)	5 (4)	
		2a	27 (15)	13 (9)	
		2b	58 (32)	33 (24)	
		2c	35 (19)	34 (24)	
		3	45 (25)	40 (29)	
	Recanalization of survivors after 3 days	0	64 (36)	55 (39)	0.381
		1	40 (22)	35 (25)	
		2	32 (18)	27 (19)	
		3	44 (24)	23 (16)	
	The final lesion volume of survivors at the discharge of hospital (mL)	Minimum	10	10	0.203
		Maximum	100	100	
		Mean ± SD	42.15 ± 13.18	40.22 ± 11.18	

Constant and ordinal variables are demonstrated as frequency (percentages) and continuous variables are demonstrated as mean ± standard deviation (SD)

Unpaired *t*-test was (for continuous variables) and Fischer exact test or the Chi-square test of Independence (for constant and ordinal variables) were performed for statistical analyses

Results were significant if *p*-value < 0.05

### Adverse effects

The most common reported adverse effect within 90 days after endovascular thrombectomy was asymptomatic intracranial hemorrhage. There were no significant differences in the reported adverse effects between both cohorts. The details of adverse effects are reported in Table 5.

**Table 5:** Adverse effects within 90 days after endovascular thrombectomy with/ without intravenous alteplase intervention

Adverse event	Cohorts		Comparisons
	EA cohort	ET cohort	
Treatment(s)	Endovascular thrombectomy + intravenous alteplase intervention	Endovascular thrombectomy	
Numbers of patients who underwent treatment	184	141	*p*-value
Asymptomatic intracranial hemorrhage	69 (38)	45 (32)	0.297
Symptomatic intracranial hemorrhage	13 (7)	5 (4)	0.171
Infarction in new territory	10 (5)	7 (5)	0.851
Large or malignant middle cerebral artery infarction	25 (14)	15 (11)	0.424
Pneumonia	15 (8)	10 (7)	0.723
Aspiration related event(s)	12 (7)	9 (6)	0.959
Allergic reaction to contrast material	2 (1)	1 (1)	0.725
Other adjudicated adverse effect(s)	15 (8)	11 (8)	0.908

Variables are demonstrated as frequency (percentages)

An unpaired *t*-test was performed for statistical analysis

Results were significant if *p*-value < 0.05

Patients might have more than one adverse effect

Heidelberg criteria

### Mortality

A total of 28 (15%) patients from the EA cohort and 24 (17%) patients from the EA cohort have died within 90 days after endovascular thrombectomy (*p* = 0.761).

## Discussion

The current study reported statistically insignificant differences for the modified Rankin scale score, procedural complications, imaging outcomes, and adverse effects of survivors within 90 days after thrombectomy and death within 90 days after thrombectomy between patients who underwent endovascular thrombectomy with intravenous alteplase intervention and those who underwent without intravenous alteplase intervention. The non-inferior outcome measures of the study were consistent with those of a randomized trial [2], the DEVT trial [8], pooled analyses [5, 6] but not consistent with those of a meta-analysis [9], a randomized trial [11], and a SKIP trial [10]. The reason for these contradictory results between the current study and those of a meta-analysis [9] and those of a SKIP trial [10] is different inclusion criteria of the studies and that for the contradictory results between the current study and those of a randomized trial [11] is unbalanced randomization of a trial. Most notably, the contraindication to alteplase would confound the cohort of patients who did not get alteplase and not allow for a clean comparison with the alteplase plus endovascular thrombectomy cohort. A brief time between administering intravenous alteplase and endovascular thrombectomy procedure did not express a significant effect of intravenous alteplase in patients [24]. The results of the current study do not rule out the benefit of intravenous alteplase intervention but patients who underwent endovascular thrombectomy without intravenous alteplase have better results than those who underwent endovascular thrombectomy with intravenous alteplase intervention.

The current study reported unfavorable effects of administering intravenous alteplase before endovascular thrombectomy on the National Institutes of Health Stroke Scale scores, the Barthel Index score, and the 5-Dimension 5-Level Self-Report Questionnaire of survivors. The inferior outcome measures of the current study were consistent with a meta-analysis [7] but not consistent with those of randomized trials [2, 11]. The large sample size (type-II error) of a randomized trial [2] (disease-oriented evidence requires smaller sample sizes for analyses [25]) and unbalanced randomization of a trial [11] are responsible for contradictory results. The outcomes of endovascular thrombectomy with or without intravenous alteplase are different in Chinese patients with acute ischemic stroke than those of white patients [16]. Administering intravenous alteplase before endovascular thrombectomy may worsen the health status of Han Chinese patients with acute ischemic stroke. However, more data and work need to be validated because 49% patients of the ET cohort had received anticoagulant medications.

The current study described a series of patients undergoing mechanical thrombectomy using the AngioJET system, which is a system used for peripheral thrombectomy. The Stroke thrombectomy systems used in all trials that established the benefits of endovascular treatment were performed with a stent- retriever or a large-bore aspiration system [26]. The technique used is not commonly used, no randomized controlled trials have used this device, and there is no validation for intracerebral use in ischemic arterial stroke. Mechanical thrombectomy has been recommended by European guidelines 7 and by the National Institute for Health and Care Excellence, and as the preferred treatment of acute ischemic stroke, particularly for large-vessel occlusions [27]. The AngioJet Thrombectomy system offers catheters for treating vessels of ≥1.5 mm clot burdens. In the current study, clots were larger, and a stent-retriever or a large-bore aspiration system is not a much effective technique when large clots are targeted [28]. Therefore, the AngioJET system was used to treat ischemic arterial stroke.

There are several limitations of the study that have to be reported, for example, retrospective study and lack of randomized trial. In the other limitations of the study, the management of acute ischemic stroke of patients was performed according to the American Heart Association-American Stroke Association guidelines [17], in which stent retrievers were performed before endovascular thrombectomy and 0.9 mg/ kg intravenous alteplase (10% bolus and 90% infusion throughout 1 h) administered. Aspiration catheters and tenecteplase were not used for the management of acute ischemic stroke patients. The results of the current study on Han Chinese patients are not generalizable to the other than Han Chinese people because there are differences in the prevalence of stroke between the Chinese population and white people. Also, the prehospital triage method is more complicated in mainland China than the Western countries. The reperfusion rate is disease-related proof [25]. Improvements in reperfusion (64% survivors from the EA cohort and 61% survivors from the ET cohort were reported partial or complete recanalization (the modified Arterial Occlusive Lesion score ≥ 1) at 3 days after endovascular thrombectomy) was reported but these did not translate into clinical benefits (patients of both cohorts reported moderate clinical outcomes; only 38% survivors from the EA cohort and 39% from survivors from the ET cohort had the modified Rankin scale score between 0 and 2 at 90 days after endovascular thrombectomy). The reasons for these are unclear. Of greater interest was the reported use of the AngioJet AVX Thrombectomy Catheter for acute ischemic stroke thrombectomy. This is not conventionally used for cerebral arterial occlusive lesions in Europe or North America because that is a pharmaco-mechanical peripheral thrombectomy device with an active aspiration that is not released for neurovascular use in stroke. This experience may be of interest, particularly in comparison to either direct aspiration or stent-retriever thrombectomy, the two primary technical approaches used. The current study compared cohorts that were treated with intravenous plus intraarterial therapy (when indicated) versus a cohort that had a contraindication for intravenous treatment (serious head trauma, prior stroke, hemorrhagic coagulopathy, or receiving anticoagulant medications). Therefore, they are different populations, and a direct comparison of results is not possible.

## Conclusions

This study attempts to address the knowledge gap regarding the impact (positive or negative) of intravenous alteplase treatment along with mechanical thrombectomy among the Han Chinese population. Endovascular thrombectomy alone treatment is statistically the same as endovascular thrombectomy with intravenous alteplase treatment regarding procedural complications, imaging outcomes, and the modified Rankin scale score in Han Chinese patients with acute ischemic stroke. Endovascular thrombectomy with intravenous alteplase treatment may have unfavorable effects on the ischemic stroke condition, functional outcome measures, and 5-Dimension 5-Level Self-Report Questionnaire of survivors during the follow-up period. However, it is appropriate to follow the American Heart Association/ American Stroke Association and the Chinese Stroke Association guidelines that patients with acute ischemic stroke should receive alteplase before endovascular thrombectomy if a patient is eligible for the same until the new recommendations. The current study adds to the current understanding of whether alteplase confers benefit before endovascular thrombectomy, which does not aim to address the other recently published studies on Han Chinese patients. However, there may be a selection bias and associated limitations of a retrospective study and needs to be validated in a large registry-based study or randomized control trial.

## Data Availability

The datasets generated during and/or analyzed during the current study are not publicly available but are available from the corresponding author on reasonable request.

## Abbreviations

EA cohort: Patients have received endovascular thrombectomy with intravenous alteplase, ET cohort Patients have been treated with endovascular thrombectomy alone
SD: Standard deviation
